# Ominous combination: COVID‐19 disease and *Candida auris* fungemia—Case report and review of the literature

**DOI:** 10.1002/ccr3.4827

**Published:** 2021-09-12

**Authors:** Wael Goravey, Gawahir A. Ali, Masia Ali, Emad B. Ibrahim, Muna Al Maslamani, Hamad Abdel Hadi

**Affiliations:** ^1^ Department of Infectious Diseases Communicable Diseases Centre Hamad Medical Corporation Doha Qatar; ^2^ Department of Laboratory Medicine and Pathology Hamad Medical Corporation Doha Qatar; ^3^ Biomedical Research Centre Qatar university Doha Qatar

**Keywords:** *Candida auris*, candidemia, COVID‐19

## Abstract

The identification of *Candida auris* fungemia in critically ill COVID‐19 patients is detrimental, with huge implications on patient mortality and infectious control measures.

## Graphical abstract

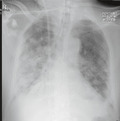

## 1 INTRODUCTION

*Candida auris* is an emerging highly resistant pathogen with considerable mortality, particularly in critically ill patients. We describe a case of fatal candidemia caused by *C. auris* in a patient with critical COVID‐19 disease to raise awareness of the circumstances leading to coinfection with this emerging resistant yeast.

The year 2019 heralds the start of severe acute respiratory coronavirus 2 (SARS‐Cov‐2) and its clinical manifestation COVID‐19 disease which initially originated from China then propagated globally leading to significant morbidity and mortality as well as considerable economic consequences.^1^ Although the disease has a wide range of clinical manifestations, only patients with severe and critical illnesses have the most serious consequences. During the course of the perplexing disease, it was noticed early, leading to ominous consequences such as the development of catastrophic immunological storms as well as acute respiratory distress syndrome (ARDS) which promoted the use of various therapeutic interventions including immunosuppressive therapy.^2^ Dysregulation of the immune responses particularly cytokine storm led to the use of steroids, interleukin (IL) inhibitors such as IL‐1 inhibitor; anakinra, and IL‐6 inhibitor; tocilizumab which compromise host immune responses provoking the emergence of latent and opportunistic coinfections including multidrug‐resistant organisms.^3^ Among secondary consequences, fungal infections with all their morbidity and mortality have been reported early during the pandemic.^4^ This can be explained because the pandemic caught all health systems around the world by surprise, escalating cases at critical care beds facilitated infection spread, invasive devices including mechanical ventilation accelerated infections risks while the use of broad‐spectrum antibiotics as well as immunosuppressive therapy, created the suitable environment for coinfections.^5^ Consequently, the advent of resistant opportunistic organisms such as *C. auris* is inevitable.^6^

*Candida auris*, an emerging multidrug‐resistant candida is first described in Japan then subsequently spread globally with the ability to cause invasive infection as well as pose substantial risks for infection control and prevention.^7^

From accumulated experience in treating patients in critical care, timely pathogen identification and early recognition of antimicrobial susceptibilities are of paramount importance as manifested in cases of invasive fungemia best outlined through multidrug‐resistant candidemia where appropriate antifungal treatment with concurrent strict infection control and prevention measures are crucial to avoid ominous outcomes.^8^

To highlight this, we describe a case of fatal *C. auris* fungemia in a critically ill COVID‐19 patient discussing relevant associated risks leading to the event aiming to raise awareness regarding the circumstances and the emergence of multidrug‐resistant opportunistic pathogens during the COVID‐19 pandemic and review the literature for similar cases.

## 2 CASE DESCRIPTION

**FIGURE 1:**
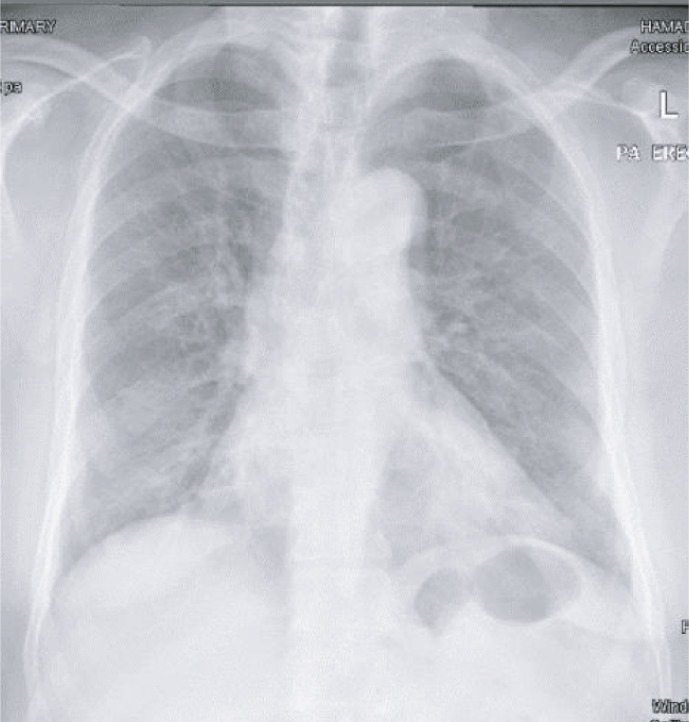
Chest X‐ray demonstrating patchy ground glass opacity in the lower lung zones bilaterally

**FIGURE 2:**
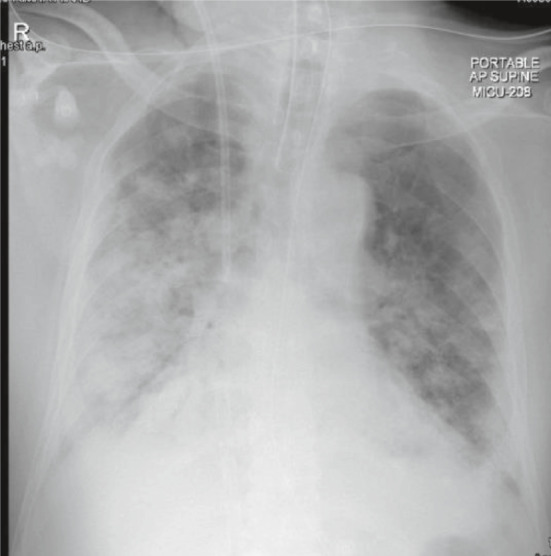
Chest X‐ray highlighting the progression of bilateral pulmonary infiltration, consolidations, and pleural effusion

A 64‐year‐old gentleman with no known comorbidities presented to our emergency department with symptoms of fever, cough, and progressive shortness of breath for about 10 days duration. Because of the typical symptoms of COVID‐19 disease in the context of the pandemic, the diagnosis was suspected early were chest X‐ray demonstrated bilateral infiltrates, and the diagnosis was subsequently confirmed by a positive SARS‐Cov2 PCR then managed as per local protocols at that time with azithromycin and a combination of lopinavir and ritonavir (Figure 1). Shortly after admission, the patient deteriorated with elevated inflammatory markers, including IL‐6 230 pg/ml (normal <8) and ferritin 2331 μ/L (30–553) progressed to ARDS, clinically assessed as COVID‐19‐related cytokine storm (CCS). The patient was admitted to the intensive care unit (ICU), intubated, and treated with steroids as well as an IL‐6 inhibitor, tocilizumab (total dose of 1200 mg, in two separate doses) in addition to broad‐spectrum antibiotics, piperacillin‐tazobactam, teicoplanin then cefepime to manage the accompanying ventilator‐associated pneumonia. Despite maximum measures of high plateau pressures and prone ventilation positions, the patient failed to maintain adequate saturation levels and continued to deteriorate which prompted commencing veno‐venous extracorporeal membrane oxygenation (ECMO). During the subsequent course, the patient received additional courses of antibiotics to treat sepsis when MRSA and *Pseudomonas aeruginosa* were isolated from tracheal aspirates. Due to worsening of the clinical condition with increased inflammatory markers, progressive radiological shadowing, and non‐response to broad‐spectrum antibiotics, bronchoscopy, and bronchoalveolar lavage performed on day 23 of ICU admission grew *Candida tropicalis* when anidulafungin was added to the management. Despite these measures, the patient continued to deteriorate developing acute kidney and liver injuries necessitating hemodialysis support. Eventually, the patient's condition stabilized and was weaned off the ventilator support through tracheostomy accompanied by the settling of radiological and inflammatory markers. However, on day 41, the patient developed fever, raised inflammatory markers, and new radiological infiltrates (Figure 2). The patient underwent repeated bronchoscopy and was covered empirically with liposomal amphotericin to cover the subsequently isolated *Candida glabrata* resistant to echinocandins. Despite initial improvement, the patient deteriorated with septic shock on day 45 with rapid deterioration and eventually succumbed to death. Subsequently, blood cultures obtained from the time of deterioration grew *C. auris* from both central and peripheral lines resistant to fluconazole and amphotericin but were susceptible to echinocandins.

## 3 DISCUSSION

After almost a century of a serious worldwide disease, the novel COVID‐19 emerging pandemic started to shape the profile of infectious diseases and health care across the globe. Undiagnosed clusters of pneumonia cases in Wuhan city, China diagnosed eventually as a novel SARS‐COV‐2 virus and COVID‐19 disease spearheaded global consequences with significant morbidity, mortality, and socioeconomic consequences never witnessed before.^1^

Because of enhanced communication and global collaborations, the clinical manifestations of the COVID‐19 disease were recognized, highlighted, and shared early together with associated infective and non‐infective consequences including secondary microbial infections particularly healthcare‐associated infections (HAIs).^9^ Of the leading HAIs, secondary fungal infections are particularly worrisome since it is usually associated with delayed diagnosis, suboptimal management with significant morbidity and mortality leading to ominous consequences. With the evolving COVID‐19 pandemic, various nosocomial invasive secondary fungal infections were inevitable spanning common organisms such as candida sp and aspergillus sp to the relatively uncommon such cryptococcal disease as well as the rare occurrence of *C. auris* ^6^, ^9^

Together with other invasive fungal infections, patients reported with invasive *C. auris* disease share many risk factors such as critical disease including recurrent sepsis, prolonged ICU stay with invasive devices, mechanical ventilation, broad‐spectrum antibiotics, and immunosuppressive conditions.^10^ Our patient fulfilled all the highlighted risk factors that favor acquiring invasive fungal disease in addition to the selective management of respiratory failure secondary to ARDS with ECMO support. It is worth noticing both ARDS and ECMO carry higher risks of secondary and opportunistic infections including invasive fungal disease.^11^ Furthermore, management of critical disease during the unprecedented pandemic, entailed unconventional approaches repurposing different modalities of therapeutic interventions. To suppress the harmful effect of the secondary immunological storm (CCS), immunosuppressive therapy such as steroids and interleukins inhibitors such as anakinra and tocilizumab has been used frequently with accumulated evidence of efficacy.^12^ It has been suggested that immunosuppressive treatment particularly biological components are associated with secondary microbial infections in mechanically ventilated patients with COVID‐19.^3^

It is plausible in our case to assume the invasive *C. auris* infection is a nosocomial infection acquired through the central line device since it has been recently recognized by the institution, worsened by the busy pandemic as well‐being isolated concomitantly peripherally and centrally from the cultured line within the conceivable time frame. From previous outbreaks and reported invasive diseases, *C. auris* has a high ability to colonize the skin and environmental surface, including medical devices, leading to significant loss of devices, nosocomial outbreaks as well as significant mortalities.^13^ The reported mortality rate of *C. auris* fungemia during the COVID‐19 disease is 60%, and surprisingly, *C. auris* was the most common cause among candida species.^10^

Regarding appropriate antifungal therapy, *C. auris* is surprisingly resistant to amphotericin therapy being sensitive to echinocandins.^14^ The clinical judgment of covering our patient with amphotericin was appropriate at the time to cover resistant *C. glabrata* grew from bronchoalveolar lavage cultures. The absence of clinical response in such cases should raise the suspicion of an alternative/and or second pathology.

The identification of *C. auris* fungemia is a crucial step in management and should be followed by actively implementing strict infectious control measures such as contact precautions, screening, and diligent decolonization of the patients.^8^ The organism is notoriously difficult to apply infection control and prevention measures once established in healthcare settings and all effort should be sought to contain the problem geographically avoiding patient's movement with the hospital as well as cross transfer to other healthcare facilities. Despite that, these infectious control measures might further jeopardize the limited intensive care beds available to critically ill patients with COVID‐19 especially in countries with limited resources.^6^

The management of *C. auris* fungemia is challenging, and multidrug‐resistant *C. auris* isolates are defined when resistance identified to the three main classes of antifungal with fluconazole yields the highest minimal inhibitory concentration(MIC) while echinocandin is an appropriate empirical first choice of therapy.^14^

Unfortunately, the blood cultures of our patient were revealed positive after he passed away due to his *C. auris* fungemia and severe COVID‐19 infection.

Our search of the literature yielded a total of 36 cases of infection due to *C. auris* in COVID‐19. (Table 1). Male constituted the majority and the age range 25–86 years. Candidemia is the predominant presentation with five cases were isolated from both urine and blood.^10,15,16^ Of note, only six cases reported of *C. aruis* in urine alone and three isolates from deep tracheal aspirated.^15,16^ Days in the hospital to develop the infection ranging between 4–45 days and most patients have significant underlining comorbidities. Almost half of the reported cases were associated with either bacterial or other fungal coinfection and/or colonization.^10,15,17^ Of the 36 cases reviewed, almost all have a urinary indwelling catheter, central lines, and on broad‐spectrum antibiotics except for four cases where the data is not obtainable.^17^ Most cases received steroids while only five cases received tocilizumab, though the data were not always available. The mortality rate is 53% of the 30 cases with the documented outcomes (Table 1).

**TABLE 1:** Summary of previously reported adult cases of infection due to *Candida auris* in COVID‐19

Study	Case	Gender/Age, years	Days in hospital	Days in hospital to positive blood/urine culture	Underlining comorbidities	Coinfection/colonization	Intubation	Urinary indwelling catheter	Presence of central lines	Broad‐spectrum antibiotics used	Steroid used	TOCI used	Anti‐fungal used	Outcome
Chowdhary, 2020^10^	1	F/25	35	14	CLD with grade II encephalopathy, AKI	4 of the 10 cases reported by Chowdhary developed bacteremia (*Enterobacter cloacae* and *Staphylococcus haemolyticus*)	Half of the cases reported by Chowdhary were received mechanical ventilation	Yes	Yes	Yes	No	No	AMB	Alive
Chowdhary, 2020^10^	2	M/52	20	14	HTN, DM	As noted above on the same study	As noted above on the same study	Yes	Yes	Yes	Yes	Yes	MFG and AMB	Died
Chowdhary, 2020^10^	3	F/82	60	42(blood and urine)	HTN, DM, hypothyroidism, on dialysis for CKD stage 5	As noted above on the same study.	As noted above on the same study	Yes	Yes	Yes	Yes	No	MFG	Died
Chowdhary, 2020^10^	4	F/86	21	10	CLD, IHD, DM	As noted above on the same study.	As noted above on the same study	Yes	Yes	Yes	Yes	No	MFG	Died
Chowdhary, 2020^10^	5	M/66	20	11	HTN, DM, asthma	As noted above on the same study	As noted above on the same study	Yes	Yes	Yes	No	No	MFG and AMB	Alive
Chowdhary, 2020^10^	6	M/71	32	12(blood and urine)	Hypothyroidism, on dialysis for CKD stage 5	As noted above on the same study	As noted above on the same study	Yes	Yes	Yes	Yes	No	MFG	Died
Chowdhary, 2020^10^	7	M/67	21	11	HTN, DM, COPD	As noted above on the same study	As noted above on the same study	Yes	Yes	Yes	Yes	No	MFG and AMB	Alive
Chowdhary, 2020^10^	8	M/72	27	16	HTN, CLD	As noted above on the same study	As noted above on the same study	Yes	Yes	No	Yes	Yes	MFG	Died
Chowdhary, 2020^10^	9	M/81	20	15	DM, HTN, IHD	As noted above on the same study	As noted above on the same study	Yes	Yes	Yes	Yes	No	MFG	Died
Chowdhary, 2020^10^	10	M/69	21	14	HTN, asthma	As noted above on the same study	As noted above on the same study	Yes	Yes	Yes	Yes	Yes	MFG	Alive
Rodriguez, 2020^18^	11–16	6 out of 20 cases reported were C. auris	Of the 20 cases,13 M and 7 F	Age mean 63	NA	Mean number of days 17.7 (range 6–35 days)	HTN, DM, CKD, Cancer	NA	19 out of the 20 cases	19 out of the 20 cases	19 out of the 20 cases	All patients received β‐lactam antibiotics	19 out of the 20 cases	30‐day mortality is 60%
Villanueva‐Lozano, 2021^15^	17	M/51	Mean 20–70 days	37	HTN, DM2, Obesity	*Pseudomonas aeruginosa*	Yes	Yes	Peripherally inserted central lines	Yes	Yes	No	CAS, ANF	Died
Villanueva‐Lozano, 2021^15^	18	M/54	Mean 20–70 days	17, urine	HTN, DM2, Obesity, Asthma	*Pseudomonas aeruginosa, Klebsiella pneumoniae*	Yes	Yes	Peripherally inserted central lines	Yes	Yes	No	ISA, CAS	Alive
Villanueva‐Lozano, 2021^15^	19	M/35	Mean 20–70 days	29	HTN, DM2, CAD	*Pseudomonas aeruginosa*, *Candida glabrata*	Yes	Yes	Peripherally inserted central lines	Yes	Yes	No	ANF	Died
Villanueva‐Lozano, 2021^15^	20	M/51	Mean 20–70 days	36, urine	Obesity	*Pseudomonas aeruginosa*, *Candida glabrata*, *Enterococcus faecalis*	Yes	Yes	Peripherally inserted central lines	Yes	Yes	No	ISA, ANF	Died
Villanueva‐Lozano, 2021^15^	21	M/64	Mean 20–70 days	13 (blood and urine)	AKI	*Pseudomonas aeruginosa*	Yes	Yes	Peripherally inserted central lines	Yes	Yes	No	CAS, VRC, AMB (intravesical)	Died
Villanueva‐Lozano, 2021^15^	22	M/64	Mean 20–70 days	10 (blood and urine)	HTN, Smoking, Obesity, Hypothyroidism	*Candida glabrata*	Yes	Yes	Peripherally inserted central lines	Yes	Yes	Yes	ANF, ISA	Died
Villanueva‐Lozano, 2021^15^	23	F/54	Mean 20–70 days	31	HTN, Obesity	None	Yes	Yes	Peripherally inserted central lines	Yes	Yes	No	AMB, CAS, VRC	died
Villanueva‐Lozano, 2021^15^	24	F/60	Mean 20–70 days	16, urine	Obesity	*Pseudomonas aeruginosa*	Yes	Yes	Peripherally inserted central lines	Yes	Yes	No	CAS, ANF, VRC	Died
Villanueva‐Lozano, 2021^15^	25	M/58	Mean 20–70 days	27, urine	HTN, Obesity	*Pseudomonas aeruginosa*	Yes	Yes	Peripherally inserted central lines	Yes	Yes	No	ANF	Died
Villanueva‐Lozano, 2021^15^	26	M/36	Mean 20–70 days	22, Urine	DM2, Obesity	*Cytomegalovirus*	Yes	Yes	Peripherally inserted central lines	Yes	Yes	Yes	CAS	Alive
Villanueva‐Lozano, 2021^15^	27	M/66	Mean 20–70 days	11, Urine	HTN, DM2, CAD, VHD	*Pseudomonas aeruginosa*, *Stenotrophomonas maltofilia*	Yes	Yes	Peripherally inserted central lines	Yes	Yes	No	VRC, CAS	Alive
Villanueva‐Lozano, 2021^15^	28	M/46	Mean 20–70 days	27	Obesity	None	Yes	Yes	Peripherally inserted central lines	Yes	Yes	No	VRC, CAS	Alive
Magnasco, 2021^17^	29	M/62	NA	45 (Time from ICU Admission to First Isolation	None	*Pseudomonas aeruginosa,*	NA	NA	NA	Yes	NA	NA	CAS	Alive
Magnasco, 2021^17^	30	M/69	NA	41 (Time from ICU Admission to First Isolation	CAD	*Pseudomonas aeruginosa*	NA	NA	NA	Yes	NA	NA	CAS, 19 days; then AMB 7 days	Died
Magnasco, 2021^17^	31	M/62	NA	26 (Time from ICU Admission to First Isolation	HTN	*Pseudomonas aeruginosa*	NA	NA	NA	Yes	NA	NA	CAS	Alive
Magnasco, 2021^17^	32	M/64	NA	4 (Time from ICU Admission to First Isolation	HTN, asthma	*Pseudomonas aeruginosa*	NA	NA	NA	Yes	NA	NA	CAS	Died
Allaw, 2021^16^	33	M/75	NA	40, Blood and urine	metastatic prostate cancer	NA	Yes	Yes	Yes	Yes	Yes	NA	Yes	Alive
Allaw, 2021^16^	34	F/68	NA	40, DTA	None	NA	Yes	Yes	Yes	Yes	Yes	NA	Yes	Alive
Allaw, 2021^16^	35	M/71	NA	15, DTA	Cutaneous T cell lymphoma in remission	NA	Yes	Yes	Yes	Yes	Yes	NA	Yes	Alive
Allaw, 2021^16^	36	M/75	NA	10, DTA	None	NA	Yes	Yes	Yes	Yes	Yes	NA	Yes	Alive
Our case,2021	37	M/64	47	45	None	MRSA, *Pseudomonas aeruginosa*, *Candida tropicalis, Candida glabrata, Trichosporon asahii*	Yes	Yes	Yes	Yes	Yes	Yes	ANF, AMB, Passed away before the final identifications	Died

Abbreviations: AKI, acute kidney injury; AMB, amphotericin B; ANF, anidulafungin; CAD, coronary artery disease; CAS: caspofungin; CKD, chronic kidney disease; CLD, chronic liver disease; COPD, chronic obstructive pulmonary disease; DM, diabetes mellitus; DTA, deep tracheal aspirate; HTN, hypertension; IHD, ischemic heart disease; ISA, isavuconazole; MFG, micafungin; TOCI, tocilizumab; VHD, valvular heart disease; VRC, voriconazole.

## 4 CONCLUSION

*Candida auris* fungemia is an emerging invasive pathogen in critically ill coronavirus disease 2019 patients that required attention, not only due to high mortality and resistance profile but to scrupulous infectious control measures to prevent the potential nosocomial spread.

## CONFLICT OF INTEREST

None declared.

## AUTHOR CONTRIBUTIONS

WG: Corresponding author, clinical management, data acquisition, literature search, manuscript writing, and final proof reading. GA: Contribution of data acquisition, manuscript preparation, and literature search. MA: Clinical management, data acquisition, and manuscript writing. EI contributed to data acquisition and Microbiology reports. HA and MA supervised all the aspects.

## CONSENT

Published with written consent of the patient.

## 5 ETHICAL APPROVAL

Ethics approval and permission were obtained to publish the case reports from the institutional review board which is in line with international standards, MRC04201075.

## Data Availability

The authors confirm that the datasets supporting the findings of this case including obtained consent from the patient`s next of kin in accordance with the journal's patient consent policy are available from the corresponding author upon request.

## References

[1] Qun L, Xuhua G, Peng W, et al. Early transmission dynamics in Wuhan, China, of novel coronavirus‐infected pneumonia. N Engl J Med. 2020;382(13):1199–1207. 31995857 10.1056/NEJMoa2001316 PMC7121484

[2] Sterne JAC, Murthy S, Diaz JV, et al. Association between administration of systemic corticosteroids and mortality among critically ill patients with COVID‐19: a meta‐analysis. JAMA. 2020;324(13):1330–1341. 32876694 10.1001/jama.2020.17023 PMC7489434

[3] Somers EC, Eschenauer GA, Troost JP, et al. Tocilizumab for treatment of mechanically ventilated patients with COVID‐19. Clin Infect Dis. 2020;73(2):e445–e454. 10.1093/cid/ciaa954 PMC7454462 32651997

[4] Chen N, Zhou M, Dong X, et al. Epidemiological and clinical characteristics of 99 cases of 2019 novel coronavirus pneumonia in Wuhan, China: a descriptive study. Lancet. 2020;395(10223):507–513. 32007143 10.1016/S0140-6736(20)30211-7 PMC7135076

[5] Antinori S, Bonazzetti C, Gubertini G, et al. Tocilizumab for cytokine storm syndrome in COVID‐19 pneumonia: an increased risk for candidemia? Autoimmun Rev. 2020;19(7):102564. 10.1016/j.autrev.2020.102564 32376396 PMC7200127

[6] Chowdhary A, Sharma A. The lurking scourge of multidrug resistant *Candida auris* in times of COVID‐19 pandemic. J Glob Antimicrob Resist. 2020;22:175–176. 32535077 10.1016/j.jgar.2020.06.003 PMC7289732

[7] Satoh K, Makimura K, Hasumi Y, Nishiyama Y, Uchida K, Yamaguchi H. *Candida auris* sp. nov., a novel ascomycetous yeast isolated from the external ear canal of an inpatient in a Japanese hospital. Microbiol Immunol. 2009;53(1):41–44. 19161556 10.1111/j.1348-0421.2008.00083.x

[8] Osei SJ. *Candida auris*, a systematic review and meta‐analysis of current updates on an emerging multidrug‐resistant pathogen. Microbiologyopen. 2018;7(4):e00578. 29345117 10.1002/mbo3.578 PMC6079168

[9] He Y, Li W, Wang Z, Chen H, Tian L, Liu D. Nosocomial infection among patients with COVID‐19: a retrospective data analysis of 918 cases from a single center in Wuhan, China. Infect Control Hosp Epidemiol. 2020;41(8):982–983. 32279676 10.1017/ice.2020.126 PMC7180328

[10] Chowdhary A, Tarai B, Singh A, Sharma A. Multidrug‐resistant *Candida auris* infections in critically Ill Coronavirus disease patients, India, April–July 2020. Emerg Infect Dis. 2020.26(11);2694–2696. 32852265 10.3201/eid2611.203504 PMC7588547

[11] Sun HY, Ko WJ, Tsai PR, et al. Infections occurring during extracorporeal membrane oxygenation use in adult patients. J Thorac Cardiovasc Surg. 2010;140(5):1125–1132. 20708754 10.1016/j.jtcvs.2010.07.017

[12] Horby PW, Campbell M, Staplin N, et al. Tocilizumab in patients admitted to hospital withcovid‐19 (recovery): preliminary results of a randomised, controlled, open‐label, platform trial. Lancet. 2021;397(10285):1637–1645. 33933206 10.1016/S0140-6736(21)00676-0 PMC8084355

[13] Chowdhary A, Sharma C, Meis JF. Candida auris: a rapidly emerging cause of hospital‐acquired multidrug‐resistant fungal infections globally. PLoS Pathog. 2017;13(5):e1006290. 28542486 10.1371/journal.ppat.1006290 PMC5436850

[14] Chowdhary A, Prakash A, Sharma C, et al. A multicentre study of antifungal susceptibility patterns among 350 *Candida auris* isolates (2009–17) in India: role of the ERG11 and FKS1 genes in azole and echinocandin resistance. J Antimicrob Chemother. 2018;73(4):891–899. 29325167 10.1093/jac/dkx480

[15] Villanueva‐Lozano H, de Treviño‐Rangel RJ, González GM, et al. Outbreak of *Candida auris* infection in a COVID‐19 hospital in Mexico. Clin Microbiol Infect. 2021;27(5):813–816. 10.1016/j.cmi.2020.12.030 PMC7835657 33429028

[16] Allaw F, Kara Zahreddine N, Ibrahim A, et al. First *Candida auris* outbreak during a covid‐19 pandemic in a tertiary‐care center in Lebanon. Pathogens. 2021;10(2):157. 33546137 10.3390/pathogens10020157 PMC7913166

[17] Magnasco L, Mikulska M, Giacobbe DR, et al. Spread of carbapenem‐resistant gram‐negatives and *Candida auris* during the covid‐19 pandemic in critically ill patients: one step back in antimicrobial stewardship? Microorganisms. 2021;9(1):95. 10.3390/microorganisms9010095 PMC7823370 33401591

[18] Rodriguez JY, Le Pape P, Lopez O, Esquea K, Labiosa AL, Alvarez‐Moreno C. *Candida auris*: a latent threat to critically ill patients with Coronavirus disease 2019. Clin Infect Dis. 2020;ciaa1595. 10.1093/cid/ciaa1595. Online ahead of print. PMC7665436 33070175

